# *Salmonella* Typhimurium Infection Reduces the Ascorbic Acid Uptake in the Intestine

**DOI:** 10.1155/2023/2629262

**Published:** 2023-01-17

**Authors:** Trevor Teafatiller, Sandeep B. Subramanya, Nils Lambrecht, Veedamali S. Subramanian

**Affiliations:** ^1^Department of Medicine, Division of Gastroenterology, University of California, Irvine, CA 92697, USA; ^2^Department of Physiology, College of Medicine and Health Sciences, United Arab Emirates University, P.O. Box-15551, Al Ain, UAE; ^3^Department of Veteran Affairs Medical Center, Long Beach, CA 90822, USA

## Abstract

*Salmonella* Typhimurium infection of the gastrointestinal tract leads to damage that compromises the integrity of the intestinal epithelium and results in enterocolitis and inflammation. *Salmonella* infection promotes the expression of inflammasome NLRP3, leading to activation and release of proinflammatory cytokines such as IL-1*β*, and the infected host often displays altered nutrient levels. To date, the effect of *Salmonella* infection and proinflammatory cytokine IL-1*β* on the intestinal uptake of ascorbic acid (AA) is unknown. Our results revealed a marked decrease in the rate of AA uptake in mouse jejunum infected with *Salmonella* wild type (WT). However, the nonpathogenic mutant (*Δ invA Δ spiB*) strain did not affect AA uptake. The decrease in AA uptake due to *Salmonella* WT infection is accompanied by significantly lower expression of mouse (m)SVCT1 protein, mRNA, and hnRNA levels. NLRP3 and IL-1*β* expression levels were markedly increased in *Salmonella*-infected mouse jejunum. IL-1*β*-exposed Caco-2 cells displayed marked inhibition in AA uptake and significantly decreased hSVCT1 expression at both protein and mRNA levels. Furthermore, the activity of the *SLC23A1* promoter was significantly inhibited by IL-1*β* exposure. In addition, GRHPR (a known SVCT1 interactor) protein and mRNA expression levels were significantly reduced in *Salmonella*-infected mouse jejunum. These results indicate that *Salmonella* infection inhibits AA absorption in mouse jejunum and IL-1*β*-exposed Caco-2 cells. The observed inhibitory effect may partially be mediated through transcriptional mechanisms.

## 1. Introduction

Vitamin C (ascorbic acid (AA)) is a powerful antioxidant that acts as a cofactor for several key biological reactions [1]. This micronutrient is not only important for the proper function of numerous enzymatic reactions, but also sustaining optimum vitamin C body homeostasis assists in the defense against certain diseases such as cataract formation, liver disease, cancer, osteoporosis, and heart disease [2–5]. The defense mechanisms can be accredited to vitamin C's antioxidant nature, which can mitigate the damaging effects of reactive oxygen species (ROS) and oxidative stress, which are generally observed in the above named diseases. Humans obtain vitamin C through dietary sources via intestinal absorption as they cannot synthesize this micronutrient *de novo*. Pronounced vitamin C deficiency is commonly observed in the elderly population, smokers, and alcoholics [6–8]. The cause of vitamin C deficiency in the conditions mentioned above is mainly attributed to the impaired absorption at the cellular level. Vitamin C deficiency is one of the contributing factors in the pathogenesis of inflammatory bowel disease (IBD, an intestinal disorder) [9–13]. Previous studies have shown that the administration of vitamin C to patients with sepsis displayed a considerable recuperation of their clinical symptoms [14–16].

Intestinal absorption of vitamin C occurs via sodium-dependent carrier-mediated activity that includes both the sodium-dependent vitamin C transporter-1 and -2 (SVCT1 (*SLC23A1*) and SVCT2 (*SLC23A2*)) [17–20]. The two vitamin C transporter isoforms share significant homology between humans and mice [21] and are differentially expressed along the intestinal tract [22]. SVCT1 is predominantly localized at the apical membrane domain, while SVCT2 is distributed at the basolateral membrane of intestinal epithelial cells, mediating vectorial AA transport [23–26].

*Salmonella enterica* serotype Typhimurium (*S.* typhimurium) is a Gram-negative, facultative intracellular bacterium that can infect a variety of hosts and causes significant morbidity and mortality across the globe [27–30]. In addition, it is the cause for ~1.35 million cases of infection, 26,500 hospital admissions, and~420 deaths annually in the United States (https://www.cdc.gov/salmonella/index.html). Salmonellosis causes approximately 20% of all typical food- and water-borne illnesses in humans and is a significant public health and economic threat worldwide. *Salmonella* infectivity in humans leads to acute gastroenteritis accompanied by watery diarrhea [31–34]. Severe cases of *Salmonella* infection alone or alongside other pathogens may also alter nutrient levels in the infected host [35]. *Salmonella* uses M cells (microfold cells) and enterocytes to enter the subepithelial compartment of the intestinal epithelium of the infected host [36, 37], where it activates the gut-associated immune system leading to the release of cytokines [35, 38]. Among the secreted cytokines, TNF*α* (tumor necrosis factor-alpha), IL-10 (interleukin 10), IL-12 (interleukin 12), and IFN*γ* (interferon-gamma) play essential roles in mediating the inflammatory reaction in the infected host [33, 39–43]. It is known that *Salmonella* infection induces the expression of inflammasomes such as NLRP3 (nucleotide-binding, oligomerization domain- (NOD-) like receptor family, pyrin domain containing 3) to release proinflammatory cytokines like IL-1*β* (interleukin 1 beta) [44–48]. Previous studies have demonstrated that proinflammatory cytokines negatively impact intestinal absorptive and secretory functions [49–52]. Additionally, studies have also demonstrated the role of *Salmonella* infection in the pathogenesis of IBD [53, 54].

Vitamin C is indispensable for normal cellular metabolic activities and immune function. In addition, humans have a limited capacity to store adequate levels of vitamin C in the body. With these factors considered, a severe and prolonged *Salmonella* infection may negatively impact vitamin C homeostasis and lead to disturbances in the nutrient levels of infected individuals. Currently, nothing is known about the consequence of *Salmonella* infection on vitamin C intestinal absorption. Therefore, in this study, we investigated whether *Salmonella* infection alters AA absorption and, if so, what are the precise molecular mechanisms involved in this process. Our findings revealed that *Salmonella* infection inhibits intestinal AA uptake, and this inhibitory effect is partially mediated through proinflammatory cytokines and the transcription of the *SLC23A1* gene.

## 2. Materials and Methods

### 2.1. Materials

American Radiolabeled Chemicals (St. Louis, MO) and PerkinElmer Inc. (Boston, MA) were the suppliers for radiolabelled [^14^C]-AA (2.8-10 mCi/mmol, radiochemical purity > 98%). The anti-*β*-actin antibody was from Santa Cruz Biotechnology (Santa Cruz, CA). In addition, western blot protocols utilized anti-rabbit IRDye-800 and anti-mouse IRDye-680 secondary antibodies sourced from LI-COR Biosciences (Lincoln, NE). The recombinant human IL-1*β* was acquired through R&D Systems, Inc. (Minneapolis, MN). Integrated DNA Technologies (San Diego, CA) supplied the individually synthesized custom oligonucleotide primers used in RT-qPCR analysis. All further chemicals, kits, and molecular biological reagents were obtained from reputable scientific manufacturers and stored appropriately to maintain substance integrity and stability.

### 2.2. Cell Culture

Caco-2 cells from human colorectal adenocarcinoma cells (ATCC, Manassas, VA) served as an intestinal epithelial *in vitro* model. They were kept in EMEM containing 10% fetal bovine serum (FBS) and antibiotics (penicillin and streptomycin) in a 37°C cell incubator with a 95% air-5% CO_2_ and high-humidity atmosphere. Confluent monolayers of Caco-2 cells were used in these investigations to determine the effect of IL-1*β* on hSVCT1 protein and mRNA expression levels as well as to assess the impact of this proinflammatory cytokine on the rate of ^14^C-AA uptake and the activity of the *SLC23A1* promoter. For the IL-1*β* studies, Caco-2 cells were serum-starved to cause synchronization by maintaining cell cultures in EMEM with only 0.5% FBS overnight before treatment.

### 2.3. Bacterial Infection

Both *Salmonella* Typhimurium (ATCC strain 14028 [55]) wild-type (WT) and nonpathogenic (*Δ invA Δ spiB*) mutant [49] were used in these investigations. LB (Luria Bertani) broth was the growth medium for overnight bacterial cultures and then PBS was used to wash the centrifuged cultures and to adjust the volume required to reach the desired concentration for oral gavage. The C57BL/6J mice were pretreated with streptomycin [49, 56] and infected using oral gavage (100 *μ*l) with either *Salmonella* (WT) or nonpathogenic *invA spiB* mutant (10^10^ bacteria/ml). The control mice were administered 100 *μ*l of PBS vehicle. The mice then were euthanized 72 h after infection, and the intestinal samples were harvested for ^14^C-AA uptake and molecular biological analysis. All mouse studies performed were reviewed and approved prior to animal use by the Department of Veterans Affairs, Long Beach, CA, Institutional Animal Care and Use Committee (IACUC).

### 2.4. ^14^C-Ascorbic Acid (AA) Uptake

Approximately 1 cm long sheets of jejunum with the submucosa intact were harvested from mice, which were then incubated in KR (Krebs-Ringer) buffer with or without unlabeled (1 mM) AA and in the presence of labeled (0.1 *μ*Ci) AA within glass test tubes immersed in a 37°C water bath for 7 min as described before [22, 51]. Monolayers of Caco-2 cells were grown to a postconfluent phase before being treated with IL-1*β* (50 ng/ml) to perform ^14^C-AA uptake [51]. Forty-eight hours later, KR buffer was used to incubate these adherent cells at 37°C with or without unlabeled (1 mM) AA and in the presence of AA (0.1 *μ*Ci) for 30 min. 1 N NaOH was then applied to the jejunum tissue or cells to cause lysis and then the samples were incubated at 80°C in a Fisher Scientific Isotemp 825F Incubator for 15 min and neutralized with 10 N HCl. A liquid scintillation counter (Beckman Coulter, Brea, CA) recorded the radiation levels of the individual uptake lysates.

### 2.5. RT-qPCR Analysis

Each cDNA template used for RT-qPCR analysis was reverse transcribed utilizing the iScript cDNA synthesis kit (Bio-Rad) and RNA isolates extracted from mouse jejunal mucosa and Caco-2 cells that were pretreated with DNase1 (Invitrogen). The assay master mixes contained iQ SYBR Green Supermix (Bio-Rad), the appropriate gene-specific forward and reverse primers, and water. Human and mouse SVCT1, NLRP3, IL-1*β*, GRHPR, and *β*-actin primers were used in these investigations (hSVCT1: For 5′-TCATCCTCTTCTCCCAGTACCT-3′ and Rev 5′-AGAGCAGCCACACGGTCAT-3′; mSVCT1: For 5′-CAGCAGGGACTTCCACCA-3′ and Rev 5′-CCACACAGGTGAAGATGGTA-3′; mNLRP3: For 5′-ATTACCCGCCCGAGAAAGG-3′ and Rev 5′-TCGCAGCAAAGATCCACACAG-3′; mIL-1*β*: For 5′-CTCTCCAGCCAAGCTTCCTTGTGC-3′ and Rev 5′- GCTCTCATCAGGACAGCCCAGGT-3′; mGRHPR: For 5′-AATTCGGATGACCCCATCC-3′ and Rev 5′-TCAGGACACCTGGCGTGTAG-3′; h*β*-actin: For 5′-CATCCTGCGTCTGGACCT-3′ and Rev 5′-TAATGTCACGCACGATTTCC-3′; and m*β*-actin: For 5′-ATCCTCTTCCTCCCTGGA-3′ and Rev 5′-TTCATGGATGCCACAGGA-3′). RT-qPCR was also utilized to measure the heterogeneous nuclear (hn) RNA expression level for mSVCT1 in mouse jejunum using cDNA and mSVCT1 hnRNA primers (mSVCT1: For 5′-GCTTCCAGGCTCTAGATGGT-3′ and Rev 5′-GGGCAAAATCTTCGTTGGGT-3′ and m*β*-actin: For 5′-AGATGACCCAGGTCAGTATC-3′ and Rev 5′-GAGCAGAAACTGCAAAGAT-3′) to amplify the appropriate nucleotides in the intron region as previously described [57]. Relative SVCT1, NLRP3, IL-1*β*, and GRHPR expression levels were normalized to Ct values of simultaneously amplified *β*-actin expression levels [22, 51].

### 2.6. Transfection and Promoter Analysis

Caco-2 cells were cotransfected with both the *SLC23A1* (Solute Carrier Family 23 Member 1) minimal promoter plasmid DNA (3 *μ*g) and pRL-TK (HSV-thymidine kinase promoter) vector (100 ng) complexed with 3 *μ*l of Lipofectamine 2000 (Invitrogen) as described [51]. Twenty-four hours later, the Caco-2 cells were incubated with IL-1*β* for 48 h, and the promoter activity was then determined [51].

### 2.7. Western Blot Analysis

Caco-2 cells or mouse jejunal mucosa total protein samples were prepared in radioimmunoprecipitation (RIPA) buffer (Sigma) as described before [22, 51]. NuPAGE 4-12% mini gels (Invitrogen) and buffers were used to separate each total protein lysate (60 *μ*g) before the proteins were transferred onto Immobilon-FL PVDF (polyvinylidene difluoride) membranes. Afterwards, hSVCT1 (1 : 200) [58] or mSVCT1 (1 : 500) [59] or GRHPR (1 : 1000) and *β*-actin (1 : 5000) primary antibodies were applied to probe the membrane, followed by three PBS-1% Tween 20 washes. Incubation with LI-COR IRDye 800CW Goat anti-Rabbit IgG and/or IRDye 680LT Goat anti-Mouse IgG secondary antibodies (1 : 30,000 dilutions) was achieved using an orbital shaker for 45 min at room temperature, followed by three PBS-1% Tween 20 washes. The fluorescent bands were analyzed to quantify protein expression using an Odyssey infrared imaging system (LI-COR Biosciences) [51].

### 2.8. Statistical Analysis

In our study, the observed data were collected from a minimum of triplicate experimental runs using different passages of cells or from at least three sets of mice. The results were validated by Student's *t*-test against a *p* value of ≤ 0.05 to determine significance. The data are represented as mean ± SEM of multiple independent experiments and are expressed as percentage of corresponding controls.

## 3. Results and Discussion

### 3.1. Effect of Salmonella Infection on Uptake of AA and mSVCT1 Expression in Mouse Jejunum

**Figure 1:**
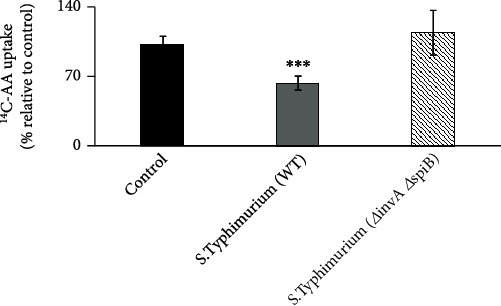
Effect of *Salmonella* infection on ^14^C-AA uptake in mouse jejunum. Streptomycin-pretreated mice were infected with *Salmonella* (WT) or mutant (*Δ invA Δ spiB*) (100 *μ*l of 10^10^ bacteria/ml) by oral gavage. ^14^C-AA uptake was subsequently determined in mouse jejunum after 72 h following oral gavage. Values are mean ± SEM of at least 3 sets of animals. ^∗∗∗^*p* < 0.001.

**Figure 2:**
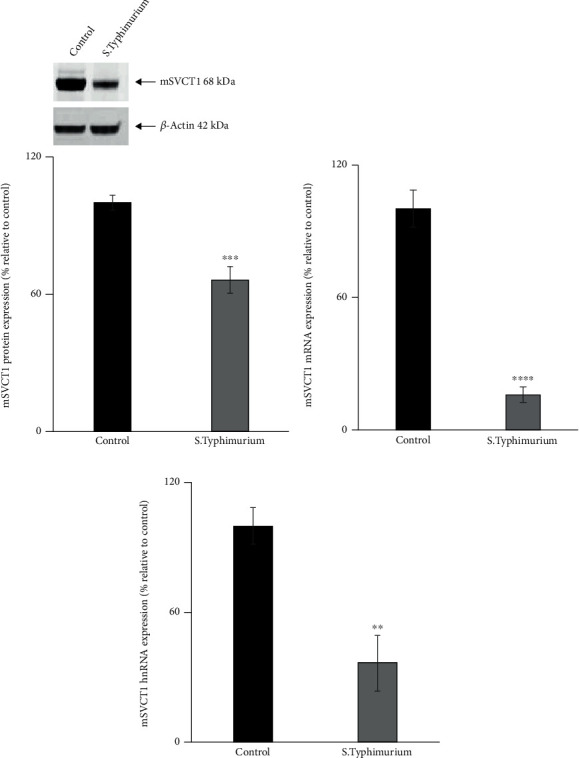
*Salmonella* infection decreases the level of expression of mSVCT1 in mouse jejunum. Western blot analysis was performed utilizing 60 *μ*g of protein isolated from *Salmonella*-exposed mouse intestine to measure the mSVCT1 protein expression (a). The mSVCT1 mRNA (b) and hnRNA (c) expression levels in mouse intestinal mucosa were measured by RT-qPCR. Values are mean ± SEM of at least 3 sets of mice. ^∗∗^*p* < 0.01, ^∗∗∗^*p* < 0.001, and ^∗∗∗∗^*p* < 0.0001.

Previous studies showed that *Salmonella* affects the intestinal mucosal physiology mainly through the immune/inflammatory response that is subsequently triggered [38, 60]. Here, we used mice as an *in vivo* model for the investigations. Streptomycin-pretreated mice were infected with *Salmonella* (WT) or nonpathogenic *Δ invA Δ spiB* mutant by oral gavage [49]. After 72 h of infection, we performed AA uptake in the jejunal sheets, which showed a significant (*p* < 0.001) decrease in the AA uptake in *Salmonella*-infected compared to uninfected control mouse jejunum (Figure 1). To further confirm that the effect of *Salmonella* infection in mice is mediated via its immune/inflammatory action, we used a nonpathogenic (avirulent) mutant (*Δ invA Δ spiB*) strain of *Salmonella* that does not cause intestinal inflammation. This mutant neither invades the intestinal mucosa nor replicates within the infected host. The nonpathogenic mutant-infected mice did not exhibit inhibition of the intestinal AA uptake (Figure 1). Therefore, we focused subsequent investigations on the inhibitory effect of the *Salmonella* (WT). Our evidence suggests that *Salmonella* infection inhibits intestinal AA uptake in mice mainly through the immune/inflammatory response that it induces [38, 60]. The observed uptake inhibition was accompanied by a marked reduction in mSVCT1 protein (Figure 2(a)), mRNA (Figure 2(b)), and hnRNA expressions (hnRNA expression levels reflect changes in the transcription rate of a given gene [57]) (Figure 2(c)) in *Salmonella*-infected compared to uninfected mouse jejunum. The latter indicates that the inhibition in SVCT1 expression in the *Salmonella*-infected mouse jejunum is partially mediated via altered transcription of the *SLC23A1* gene. Future studies will confirm the observed decreased mSVCT1 expression levels in the isolated enterocytes.

### 3.2. Effect of Salmonella Infection on NLRP3 and IL-1*β* Expression Levels in Mouse Jejunum

**Figure 3:**
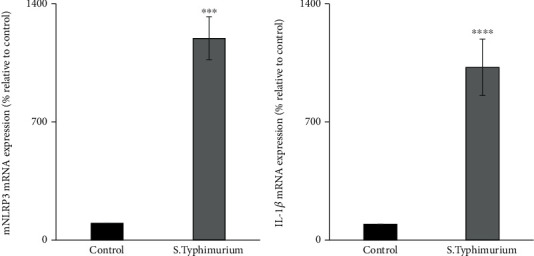
Effect of *Salmonella* infection on the expression of NLRP3 and IL-1*β* mRNA in mouse jejunum. The expression of NLRP3 (a) and IL-1*β* (b) mRNA levels in mouse jejunum by RT-qPCR. Values are mean ± SEM of at least 3 sets of animals. ^∗∗∗^*p* < 0.001 and ^∗∗∗∗^*p* < 0.0001.

Previous studies have shown that *Salmonella* infection triggers inflammasome assembly through identification by cytoplasmic receptors such as NLRP3 to release proinflammatory cytokines such as IL-1*β* [44–47]. To test this, we determined NLRP3 mRNA expression levels in *Salmonella*-infected mouse jejunum. The results showed that the NLRP3 mRNA expression was significantly (*p* < 0.001) upregulated in *Salmonella*-infected mice jejunum (Figure 3(a)) suggesting an activated inflammatory response upon *Salmonella* infection in the intestine. To substantiate this finding, we also determined the expression level of IL-1*β* in *Salmonella-*infected mouse jejunum and found significantly (*p* < 0.0001) increased IL-1*β* mRNA expression in *Salmonella*-infected mouse jejunum compared to uninfected mice (Figure 3(b)).

### 3.3. Effect of IL-1*β* on AA Uptake, hSVCT1 Expression, and the Activity of SLC23A1 Promoter in Intestinal Epithelial Caco-2 Cells

**Figure 4:**
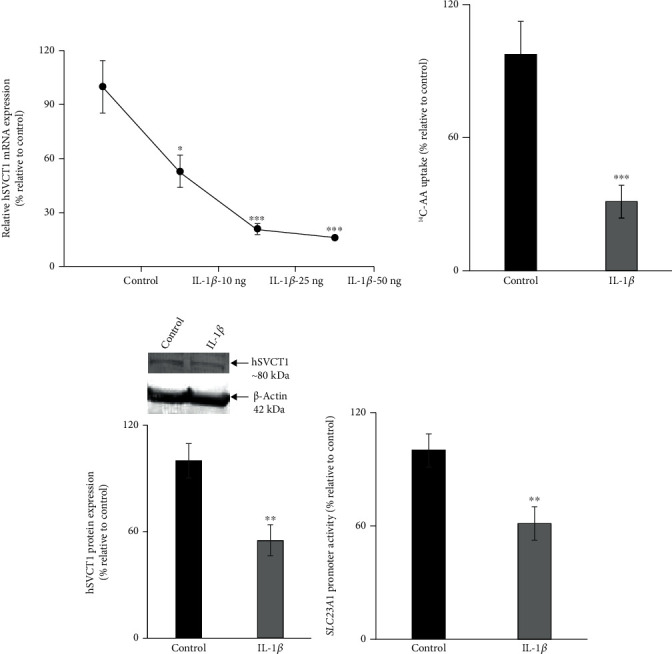
Effect of IL-1*β* on AA uptake, hSVCT1 expression levels, and the activity of *SLC23A1* promoter in Caco-2 cells. Concentration-dependent effect of IL-1*β* treatment on the hSVCT1 mRNA expression (a). Determined AA uptake in IL-1*β* (50 ng/ml)-exposed and control cells (b). Performed western blotting to quantify the expression of hSVCT1 protein in IL-1*β*-exposed and control cells (c). The activity of *SLC23A1* promoter was determined in IL-1*β*-treated and control cells (d). Values are mean ± SEM of at least 3-4 independent experimental runs using different passage of cells. ^∗^*p* < 0.05, ^∗∗^*p* < 0.01, and ^∗∗∗^*p* < 0.001.

The effect of IL-1*β* on the uptake of AA in Caco-2 cells has never been investigated before. Therefore, Caco-2 cells were exposed to three different concentrations of IL-1*β* (10, 25, and 50 ng/ml) for 48 h, and hSVCT1 mRNA expression levels were determined. The results revealed a marked decrease in hSVCT1 mRNA expression at all three IL-1*β* concentrations compared to control cells (Figure 4(a)). Subsequently, we examined the effect of IL-1*β* treatment (50 ng/ml for 48 h) on the uptake of AA in Caco-2 cells. The outcomes exhibited a marked inhibitory effect on the uptake of AA in cells exposed to IL-1*β* (Figure 4(b)). This inhibitory effect was accompanied by a marked reduction in the expression level of hSVCT1 protein (Figure 4(c)).

Furthermore, to investigate the molecular mechanism(s) involved in AA uptake inhibition upon IL-1*β* exposure, we determined the effect of IL-1*β* treatment on the *SLC23A1* promoter activity. The results displayed a marked decrease in the *SLC23A1* promoter activity in IL-1*β*-exposed cells (Figure 4(d)). Collectively, these results indicate that the inhibition of AA uptake mediated by IL-1*β* exposure is partially facilitated through transcriptional mechanism(s) involving the *SLC23A1* gene. The observed decreased expression of hSVCT1 caused by IL-1*β* is not a universal phenomenon among members of the solute carrier (SLC) superfamily of transporter proteins. Previous studies have shown that the PepT1 (peptide transporter 1, product of the*SLC15A1* gene) was upregulated upon cytokine treatment [61, 62]. Together, our findings suggest that *Salmonella*-triggered inflammasome assembly via recognition by NLRP3 induces proinflammatory cytokines such as IL-1*β*, which causes an inhibitory effect on the uptake of AA in intestinal epithelial cells.

### 3.4. Salmonella Infection Reduces the Expression of GRHPR in Mouse Jejunum

**Figure 5:**
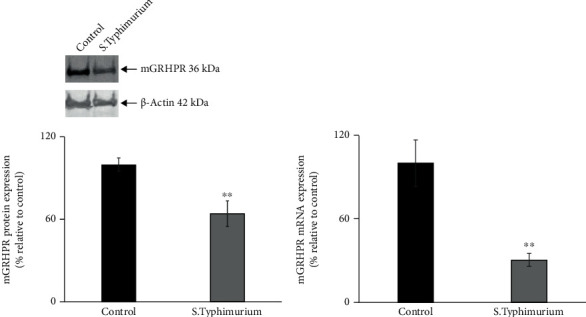
*Salmonella* infection decreases the expression of GRHPR in mouse jejunum. The GRHPR protein (a) and mRNA (b) expression levels in mouse jejunum were calculated from western blot and RT-qPCR data, respectively. Values are mean ± SEM of at least 3 sets of animal. ^∗∗^*p* < 0.01.

Previously, we have identified GRHPR (glyoxalate reductase/hydroxypyruvate reductase) as an interacting partner for SVCT1, and this association upregulates AA uptake [63]. In this study, we have determined the role of GRHPR in *Salmonella*-infected AA uptake in mouse jejunum. *Salmonella* infection triggered a marked decrease in GRHPR protein and mRNA expression in mouse jejunum (Figures 5(a) and 5(b)). These findings show that GRHPR may also play a role in the observed inhibitory effect on AA uptake in mouse jejunum.

In conclusion, our investigations demonstrate that *Salmonella* infection decreases intestinal AA absorption, and this inhibitory effect is facilitated partially through proinflammatory cytokines which are induced as a result of *Salmonella* infection in aninfected host. In addition, the observed intestinal AA uptake inhibition caused by proinflammatory cytokines may provide a novel hypothesis to explain the suboptimal levels of vitamin C observed in IBD patients [9, 12, 13].

## Data Availability

The data that support the findings of this study are available from the corresponding author upon reasonable request.

## References

[1] Packer L., Fuchs J. (1997). *Vitamin C in Health and Disease*.

[2] Carr A. C., Frei B. (1999). Toward a new recommended dietary allowance for vitamin C based on antioxidant and health effects in humans. *The American Journal of Clinical Nutrition*.

[3] Rondanelli M., Opizzi A., Perna S., Faliva M. A. (2013). Actualizacion sobre nutrientes implicados en el mantenimiento del hueso sano. *Endocrinología y Nutrición*.

[4] Simon J. A., Hudes E. S. (2000). Serum ascorbic acid and gallbladder disease prevalence among US adults: the Third National Health and Nutrition Examination Survey (NHANES III). *Archives of Internal Medicine*.

[5] Harrison S. A., Torgerson S., Hayashi P., Ward J., Schenker S. (2003). Vitamin E and vitamin C treatment improves fibrosis in patients with nonalcoholic steatohepatitis. *The American Journal of Gastroenterology*.

[6] Alcantara-Martos T., Delgado-Martinez A. D., Vega M. V., Carrascal M. T., Munuera-Martinez L. (2007). Effect of vitamin C on fracture healing in elderly osteogenic disorder Shionogi rats. *Journal of Bone and Joint Surgery. British Volume (London)*.

[7] Schectman G., Byrd J. C., Gruchow H. W. (1989). The influence of smoking on vitamin C status in adults. *American Journal of Public Health*.

[8] Schleicher R. L., Carroll M. D., Ford E. S., Lacher D. A. (2009). Serum vitamin C and the prevalence of vitamin C deficiency in the United States: 2003-2004 National Health and Nutrition Examination Survey (NHANES). *The American Journal of Clinical Nutrition*.

[9] Buffinton G. D., Doe W. F. (1995). Altered ascorbic acid status in the mucosa from inflammatory bowel disease patients. *Free Radical Research*.

[10] Hengstermann S., Valentini L., Schaper L. (2008). Altered status of antioxidant vitamins and fatty acids in patients with inactive inflammatory bowel disease. *Clinical Nutrition*.

[11] Bulger E. M., Helton W. S. (1998). Nutrient antioxidants in gastrointestinal diseases. *Gastroenterology Clinics of North America*.

[12] Buffinton G. D., Doe W. F. (1995). Depleted mucosal antioxidant defences in inflammatory bowel disease. *Free Radical Biology & Medicine*.

[13] Dunleavy K. A., Ungaro R. C., Manning L., Gold S., Novak J., Colombel J. F. (2021). Vitamin C deficiency in inflammatory bowel disease: the forgotten micronutrient. *The Forgotten Micronutrient*.

[14] Mitchell A. B., Ryan T. E., Gillion A. R., Wells L. D., Muthiah M. P. (2020). Vitamin C and thiamine for sepsis and septic shock. *The American Journal of Medicine*.

[15] Wang Y., Lin H., Lin B. W., Lin J. D. (2019). Effects of different ascorbic acid doses on the mortality of critically ill patients: a meta-analysis. *Annals of Intensive Care*.

[16] Marik P. E., Khangoora V., Rivera R., Hooper M. H., Catravas J. (2017). Hydrocortisone, vitamin C, and thiamine for the treatment of severe sepsis and septic shock: a retrospective before-after study. *Chest*.

[17] Daruwala R., Song J., Koh W. S., Rumsey S. C., Levine M. (1999). Cloning and functional characterization of the human sodium-dependent vitamin C transporters hSVCT1 and hSVCT2. *FEBS Letters*.

[18] Rajan D. P., Huang W., Dutta B. (1999). Human placental sodium-dependent vitamin C transporter (SVCT2): molecular cloning and transport function. *Biochemical and Biophysical Research Communications*.

[19] Wang Y., Mackenzie B., Tsukaguchi H., Weremowicz S., Morton C. C., Hediger M. A. (2000). Human vitamin C (L-ascorbic acid) transporter SVCT1. *Biochemical and Biophysical Research Communications*.

[20] Wang H., Dutta B., Huang W. (1999). Human Na^+^-dependent vitamin C transporter 1 (hSVCT1): primary structure, functional characteristics and evidence for a non- functional splice variant. *Biochimica et Biophysica Acta*.

[21] Savini I., Rossi A., Pierro C., Avigliano L., Catani M. V. (2008). SVCT1 and SVCT2: key proteins for vitamin C uptake. *Amino Acids*.

[22] Subramanian V. S., Srinivasan P., Wildman A. J., Marchant J. S., Said H. M. (2017). Molecular mechanism(s) involved in differential expression of vitamin C transporters along the intestinal tract. *American Journal of Physiology. Gastrointestinal and Liver Physiology*.

[23] Maulén N. P., Henríquez E. A., Kempe S. (2003). Up-regulation and polarized expression of the sodium-ascorbic acid transporter SVCT1 in post-confluent differentiated CaCo-2 cells. *The Journal of Biological Chemistry*.

[24] Boyer J. C., Campbell C. E., Sigurdson W. J., Kuo S. M. (2005). Polarized localization of vitamin C transporters, SVCT1 and SVCT2, in epithelial cells. *Biochemical and Biophysical Research Communications*.

[25] Subramanian V. S., Marchant J. S., Boulware M. J., Said H. M. (2004). A C-terminal region dictates the apical plasma membrane targeting of the human sodium-dependent vitamin C transporter-1 in polarized epithelia. *The Journal of Biological Chemistry*.

[26] Ulloa V., Saldivia N., Ferrada L. (2019). Basal sodium-dependent vitamin C transporter 2 polarization in choroid plexus explant cells in normal or scorbutic conditions. *Scientific Reports*.

[27] Brown M., Eykyn S. J. (2000). Non-typhoidal Salmonella bacteraemia without gastroenteritis: a marker of underlying immunosuppression. Review of cases at St. Thomas' Hospital 1970-1999. *The Journal of Infection*.

[28] Crump J. A., Mintz E. D. (2010). Global trends in typhoid and paratyphoid fever. *Clinical Infectious Diseases*.

[29] Feasey N. A., Dougan G., Kingsley R. A., Heyderman R. S., Gordon M. A. (2012). Invasive non-typhoidal salmonella disease: an emerging and neglected tropical disease in Africa. *The Lancet*.

[30] de Jong H. K., Parry C. M., van der Poll T., Wiersinga W. J. (2012). Host-pathogen interaction in invasive salmonellosis. *PLoS Pathogens*.

[31] Coburn B., Grassl G. A., Finlay B. B. (2007). Salmonella, the host and disease: a brief review. *Immunology and Cell Biology*.

[32] McGovern V. J., Slavutin L. J. (1979). Pathology of salmonella colitis. *The American Journal of Surgical Pathology*.

[33] Stoycheva M., Murdjeva M. (2005). Serum levels of interferon-gamma, interleukin-12, tumour necrosis factor-alpha, and interleukin-10, and bacterial clearance in patients with gastroenteric Salmonella infection. *Scandinavian Journal of Infectious Diseases*.

[34] Zhang S., Kingsley R. A., Santos R. L. (2003). Molecular pathogenesis of Salmonella enterica serotype typhimurium-induced diarrhea. *Infection and Immunity*.

[35] Eckmann L., Kagnoff M. F. (2001). Cytokines in host defense against Salmonella. *Microbes and Infection*.

[36] George J. J., Martin-Diaz L., Ojanen M. J. T., Gasa R., Pesu M., Viiri K. (2021). PRC2 regulated Atoh8 is a regulator of intestinal microfold cell (M cell) Differentiation. *Differentiation*.

[37] Balic A., Chintoan-Uta C., Vohra P. (2019). Antigen sampling CSF1R-expressing epithelial cells are the functional equivalents of mammalian M cells in the avian follicle-associated epithelium. *Frontiers in Immunology*.

[38] Raffatellu M., Santos R. L., Verhoeven D. E. (2008). Simian immunodeficiency virus-induced mucosal interleukin-17 deficiency promotes Salmonella dissemination from the gut. *Nature Medicine*.

[39] Jotwani R., Tanaka Y., Watanabe K., Tanaka K., Kato N., Ueno K. (1995). Cytokine stimulation during Salmonella typhimurium sepsis in Itys mice. *Journal of Medical Microbiology*.

[40] Arnold J. W., Klimpel G. R., Niesel D. W. (1993). Tumor necrosis factor (TNF*α*) regulates intestinal mucus production during Salmonellosis. *Cellular Immunology*.

[41] Klimpel G. R., Asuncion M., Haithcoat J., Niesel D. W. (1995). Cholera toxin and Salmonella typhimurium induce different cytokine profiles in the gastrointestinal tract. *Infection and Immunity*.

[42] Arnold J. W., Niesel D. W., Annable C. R. (1993). Tumor necrosis factor-alpha mediates the early pathology in Salmonella infection of the gastrointestinal tract. *Microbial Pathogenesis*.

[43] Pietilä T. E., Veckman V., Kyllönen P., Lähteenmäki K., Korhonen T. K., Julkunen I. (2005). Activation, cytokine production, and intracellular survival of bacteria in Salmonella-infected human monocyte-derived macrophages and dendritic cells. *Journal of Leukocyte Biology*.

[44] Bierschenk D., Boucher D., Schroder K. (2017). Salmonella-induced inflammasome activation in humans. *Molecular Immunology*.

[45] De Jong H. K., Koh G. C., van Lieshout M. H. (2014). Limited role for ASC and NLRP3 during in vivo Salmonella typhimurium infection. *BMC Immunology*.

[46] Li Y., Liu M., Zuo Z. (2017). TLR9 regulates the NF-*κ*B-NLRP3-IL-1*β* pathway negatively in Salmonella-induced NKG2D-mediated intestinal inflammation. *Journal of Immunology*.

[47] Broz P., Newton K., Lamkanfi M., Mariathasan S., Dixit V. M., Monack D. M. (2010). Redundant roles for inflammasome receptors NLRP3 and NLRC4 in host defense against Salmonella. *The Journal of Experimental Medicine*.

[48] Zaki M. H., Man S. M., Vogel P., Lamkanfi M., Kanneganti T. D. (2014). Salmonella exploits NLRP12-dependent innate immune signaling to suppress host defenses during infection. *Proceedings of the National Academy of Sciences of the United States of America*.

[49] Ghosal A., Jellbauer S., Kapadia R., Raffatellu M., Said H. M. (2015). Salmonella infection inhibits intestinal biotin transport: cellular and molecular mechanisms. *American Journal of Physiology. Gastrointestinal and Liver Physiology*.

[50] Yrlid U., Svensson M., Johansson C., Wick M. J. (2000). Salmonella infection of bone marrow-derived macrophages and dendritic cells: influence on antigen presentation and initiating an immune response. *FEMS Immunology and Medical Microbiology*.

[51] Subramanian V. S., Sabui S., Subramenium G. A., Marchant J. S., Said H. M. (2018). Tumor necrosis factor alpha reduces intestinal vitamin C uptake: a role for NF-*κ*B-mediated signaling. *American Journal of Physiology. Gastrointestinal and Liver Physiology*.

[52] Anandam K. Y., Alwan O. A., Subramanian V. S., Srinivasan P., Kapadia R., Said H. M. (2018). Effect of the proinflammatory cytokine TNF-*α* on intestinal riboflavin uptake: inhibition mediated via transcriptional mechanism(s). *American Journal of Physiology. Cell Physiology*.

[53] Axelrad J. E., Cadwell K. H., Colombel J. F., Shah S. C. (2020). Systematic review: gastrointestinal infection and incident inflammatory bowel disease. *Alimentary Pharmacology & Therapeutics*.

[54] Axelrad J. E., Olén O., Askling J. (2019). Gastrointestinal infection increases odds of inflammatory bowel disease in a nationwide case-control study. *Clinical Gastroenterology and Hepatology*.

[55] Kingsley R. A., Humphries A. D., Weening E. H. (2003). Molecular and phenotypic analysis of the CS54 island of Salmonella enterica serotype typhimurium: identification of intestinal colonization and persistence determinants. *Infection and Immunity*.

[56] Barthel M., Hapfelmeier S., Quintanilla-Martínez L. (2003). Pretreatment of mice with streptomycin provides a Salmonella enterica serovar typhimurium colitis model that allows analysis of both pathogen and host. *Infection and Immunity*.

[57] Elferink C. J., Reiners J. J. (1996). Quantitative RT-PCR on CYP1A1 heterogeneous nuclear RNA: a surrogate for the in vitro transcription run-on assay. *BioTechniques*.

[58] Heskett C. W., Teafatiller T., Hennessey C. (2021). Enteropathogenic Escherichia coli infection inhibits intestinal ascorbic acid uptake via dysregulation of its transporter expression. *Digestive Diseases and Sciences*.

[59] Subramanian V. S., Sabui S., Moradi H., Marchant J. S., Said H. M. (2018). Inhibition of intestinal ascorbic acid uptake by lipopolysaccharide is mediated via transcriptional mechanisms. *Biochimica et Biophysica Acta - Biomembranes*.

[60] Santos R. L., Raffatellu M., Bevins C. L. (2009). Life in the inflamed intestine, Salmonella style. *Trends in Microbiology*.

[61] Buyse M., Charrier L., Sitaraman S., Gewirtz A., Merlin D. (2003). Interferon-*γ* increases hPepT1-mediated uptake of di-tripeptides including the bacterial tripeptide fMLP in polarized intestinal epithelia. *The American Journal of Pathology*.

[62] Vavricka S. R., Musch M. W., Fujiya M. (2006). Tumor necrosis factor-*α* and interferon-*γ* increase PepT1 expression and activity in the human colon carcinoma cell line Caco-2/bbe and in mouse intestine. *Pflügers Archiv*.

[63] Subramanian V. S., Nabokina S. M., Patton J. R., Marchant J. S., Moradi H., Said H. M. (2013). Glyoxalate reductase/hydroxypyruvate reductase interacts with the sodium-dependent vitamin C transporter-1 to regulate cellular vitamin C homeostasis. *American Journal of Physiology. Gastrointestinal and Liver Physiology*.

